# Omicron neutralization character in patients with breast cancer and liver cancer after the nationwide omicron outbreak

**DOI:** 10.1002/cam4.7304

**Published:** 2024-06-03

**Authors:** Shaohua Zhang, Lili Tang, Chunmei Bao, Siyu Wang, Bo Li, Lei Huang, Hua Song, Junliang Fu, Zhe Xu, Fanping Meng, Lin Cao, Yingying Gao, Yue Yuan, Yangliu Chen, Jinhong Yuan, Chunbao Zhou, Fan Li, Lili Qin, Yingfei Guo, Chao Zhang, Jinwen Song, Xing Fan, Zefei Jiang, Fu‐Sheng Wang, Ruonan Xu

**Affiliations:** ^1^ Department of Medical Oncology The Fifth Medical Center of Chinese PLA General Hospital Beijing China; ^2^ Department of Infectious Diseases The Fifth Medical Center of Chinese PLA General Hospital Beijing China; ^3^ Peking University 302 Clinical Medical School Beijing China; ^4^ Southern Medical District of Chinese PLA General Hospital Beijing China

**Keywords:** antibody, cancer patients, COVID‐19, omicron, vaccine

## Abstract

**Background:**

The surge in omicron variants has caused nationwide breakthrough infections in mainland China since the December 2022. In this study, we report the neutralization profiles of serum samples from the patients with breast cancer and the patients with liver cancer who had contracted subvariant breakthrough infections.

**Methods:**

In this real‐world study, we enrolled 143 COVID‐19‐vaccinated (81 and 62 patients with breast and liver cancers) and 105 unvaccinated patients with cancer (58 and 47 patients with breast and liver cancers) after omicron infection. Anti‐spike receptor binding domain (RBD) IgGs and 50% pseudovirus neutralization titer (pVNT_50_) for the preceding (wild type), circulating omicron (BA.4‐BA.5, and BF.7), and new subvariants (XBB.1.5) were comprehensively analyzed.

**Results:**

Patients with liver cancer receiving booster doses had higher levels of anti‐spike RBD IgG against circulating omicron (BA.4‐BA.5, and BF.7) and a novel subvariant (XBB.1.5) compared to patients with breast cancer after breakthrough infection. Additionally, all vaccinated patients produced higher neutralizing antibody titers against circulating omicron (BA.4‐BA.5, and BF.7) compared to unvaccinated patients. However, the unvaccinated patients produced higher neutralizing antibody against XBB.1.5 than vaccinated patients after Omicron infection, with this trend being more pronounced in breast cancer than in liver cancer patients. Moreover, we found that there was no correlation between anti‐spike RBD IgG against wildtype virus and the neutralizing antibody titer, but a positive correlation between anti‐spike RBD IgG and the neutralizing antibody against XBB.1.5 was found in unvaccinated patients.

**Conclusion:**

Our study found that there may be differences in vaccine response and protective effect against COVID‐19 infection in patients with liver and breast cancer. Therefore, we recommend that COVID‐19 vaccine strategies should be optimized based on vaccine components and immunology profiles of different patients with cancer.

## 1 INTRODUCTION

In the last 3 years, severe acute respiratory syndrome coronavirus 2 (SARS‐CoV‐2) has caused >770 million infections and >6.95 million deaths.^1^ In the winter of 2022, the omicron variant became the dominant epidemic strain, causing nationwide breakthrough infections due to attenuated immunity and immune evasion.^2^, ^3^, ^4^ In clinics, omicron infection is characterized by less pathogenicity, milder clinical symptoms, and lower mortality risk than previous outbreaks. During the 2022 winter, the omicron outbreak rapidly increased the number of infected patients.^5^, ^6^

Vaccines have been reported to alleviate disease severity and shorten nucleic acid conversion time.^7^, ^8^ Different coronavirus disease (COVID‐19) vaccines, including inactivated, recombinant spike protein subunit, and mRNA vaccines, have been available previously in China.^9^, ^10^, ^11^ Recently, cross‐neutralization against emerging subvariants, including BQ.1, BQ.1.1, and XBB.1.5, was better in the protein subunit and omicron chimeric receptor‐binding domain (RBD)‐dimer‐vaccinated healthy controls after breakthrough infection, whereas cross‐neutralization was lower in the ancestral inactivated vaccinated controls after breakthrough infection.^12^, ^13^, ^14^, ^15^ The inactivated vaccine has been publicly available for the past 3 years. However, people with poor immune status were more likely to have vaccine reluctance or refusal for fear of adverse reactions and effectiveness.^16^, ^17^, ^18^ During the omicron surge, the omicron neutralization characteristics of these populations need to be investigated.

Patients with cancer are vulnerable populations at a high risk of mortality upon ancestral SARS‐CoV‐2 infection and should be prioritized for COVID‐19 vaccines.^18^, ^19^ Especially those patients with hematologic malignancies have impaired B cell maturation alongside the over‐activation of T cells, resulting in a poorer response to vaccines than other solid tumors. For patients with breast cancer, although CD4^+^ T cells counts are equal or even higher than those healthy individuals, the CD3^+^ T cells and CD4^+^ T cells gradually decrease with the lymphatic metastases.^20^, ^21^ In patients with hepatitis B virus (HBV)‐associated primary liver cancer, the counts of T lymphocytes, natural killer cells, B cells, CD4^+^ T cells and CD8^+^ T cells were all decreased compared to those chronic hepatitis B infection.^22^ Therefore, different types of tumor patients are characterized by different immune profiles, and immune evaluation before vaccination were needed.

Simultaneously, tumor disease stage and current treatments have been associated with the severity of COVID‐19 infection, indicating that the clinical and biochemical characteristics of different types of cancer may influence the response to the Omicron.^23^, ^24^, ^25^

In this real‐world study, we concentrated on serum samples from patients with cancer collected between February 15, 2023 and March 15, 2023. Serum sample neutralization for anti‐spike RBD IgGs and 50% inhibiting concentration for preceding (wild‐type), circulating Omicron (BA.4‐BA.5, and BF.7) and a new subvariant (XBB.1.5) were comprehensively analyzed in patients with breast and liver cancers. Finally, the factors that influenced the efficacy of the COVID‐19 vaccine in the two types of patients with cancer were analyzed, aiming to determine omicron neutralization from different unvaccinated and vaccinated patients with cancer after breakthrough infection.

## 2 METHODS

### 2.1 Study design and patient enrollment

We enrolled a section cohort of patients with cancer, mainly patients with breast and liver cancers who received a vaccine before having an omicron subvariant infection during the latest BF.7 and BA.5.2 waves in China in December 2022. The patients were admitted to the Fifth Medical Center of the Chinese PLA General Hospital for cancer treatment between February 15, 2023 and March 15, 2023. Finally, 58 and 81 unvaccinated and vaccinated patients with breast cancer, respectively, and 47 unvaccinated and 62 unvaccinated and vaccinated patients with liver cancer, respectively, were included (Figure S1). All patients with cancer were infected during the omicron surge in December 2022 and were diagnosed with mild or moderate COVID‐19 disease. The diagnostic and classification criteria for coronavirus infection were based on the “Guidelines for Prevention and Control of Coronavirus Disease 2019 (Version 9).”^26^ All patients signed informed consent. This study was approved by the Ethics Committee of the Fifth Medical Center of the Chinese PLA General Hospital (ethical approval no. ky‐2021‐7‐9‐1).

### 2.2 Clinical and laboratory parameters collection

We collected data on demographics, comorbidities, vaccinations, treatments, tumor node metastasis (TNM) stage, and lymphocyte counts at admission for cancer treatment at our center from the patients' medical records.^27^ Two other physicians checked all data separately, and a third researcher resolved any differences in interpretation between the two reviewers.

### 2.3 Definitions of vaccination

The vaccination status was categorized into three groups: unvaccinated, fully vaccinated, and booster doses. The vaccine administered to patients in our study was mainly inactivated vaccine. Patients who received two doses of the inactivated vaccine were defined as fully vaccinated, and those who received three doses of the inactivated vaccine were defined as having received a booster dose. The interval between the last dose and breakthrough infection and the time from COVID‐19 infection to hospitalization were counted separately.

### 2.4 Serum samples collection for anti‐spike RBD IgGs and 50% inhibiting concentration assay

In the section cohort, serum samples were collected 70–80 days after the surge of Omicron infection in the winter of 2022. They were classified into three groups based on the vaccination strategy: unvaccinated, fully vaccinated, and booster doses.

The pseudotyped virus neutralization assay was similar to those of previous reports,^28^ and the neutralization ability of serum samples against preceding (wild‐type), circulating Omicron (BA.4‐BA.5 and BF.7) and a new subvariant (XBB.1.5) were measured. Briefly, in this experiment, the pseudovirus neutralization detection method was used to use an HIV lentiviral vector, a pseudovirus based on the surface expression of SARS‐CoV‐2 S protein, which can bind to highly‐expressed angiotensin‐converting enzyme 2 (ACE2) of the cells and invade and integrate genes into the cells. The pseudoviral gene was modified to carry the luciferase reporter gene, which can express the luciferase protein, and can emit light color by adding the corresponding luciferase substrate to the detection system. Finally, the chemiluminescence detection function module of the microplate reader was used to detect the chemiluminescence signal value (RLU), the detection wavelength was set to full wavelength, and the reading time was set to 500 ms. The relative luminescence signal value (RLU) is inversely proportional to the amount of anti‐SARS‐CoV‐2 S protein‐neutralizing antibodies in the sample to be tested. Evaluation of pseudovirus‐infected cell activity was performed by detecting luciferase activity. Enzyme‐linked immunosorbent assay (ELISA) was used to quantitatively detect s‐IgG antibodies against SARS‐CoV‐2 Spike RBD (original and Omicron BA.4‐BA.5, BF.7, BA.4.6, and XBB.1.5 strains) protein in human plasma using previously‐described quantitative methods.^29^

### 2.5 Statistics

All statistical analyses were performed using the SPSS Statistics 24. Data were visualized using GraphPad Prism 8. Categorical variables are presented as counts and percentages, and continuous variables are presented as mean ± standard deviation. Fisher's exact test and χ^2^ test were used to compare categorical variables. Analysis of variance was used to compare continuous variables. Statistical significance was set at *p* < 0.05. Multivariate analysis was performed using a liner regression model to identify the factors that may be associated with anti‐spike RBD IgGs and 50% pseudovirus neutralization titer (pVNT_50_). All potential variables were screened before the multivariate analysis. All qualified variables were subjected to single‐factor regression, and variables with *p* < 0.1 were included in the subsequent multivariable regression analysis. The variance inflation factor was calculated for each iteration to avoid multicollinearity. Due to the strong correlation between sex and tumor type, sex variables were excluded from the multivariate analysis.

## 3 RESULTS

### 3.1 Demographic characteristics of patients

In our cohort, we included 58 unvaccinated and 81 vaccinated patients with breast cancer, 47 unvaccinated and 62 vaccinated patients with liver cancer. Data on age, sex, BMI, comorbidities, cancer disease status, and antitumor treatments were collected (Table 1). In addition, we also recorded the vaccinations, disease severity of omicron infection, clinical symptoms, antiviral treatment and the date of infection (Table 1).

**TABLE 1:** The demographic characteristics of patients.

	Breast cancer			Liver cancer		
	Un‐vaccinated (*n* = 58)	Vaccinated (*n* = 81)	*p*‐Value	Un‐vaccinated (*n* = 47)	Vaccinated (*n* = 62)	*p*‐Value
Age, years, mean (SD)	52.5 (10.1)*	51.4 (10.7)*	**0.539**	57.7 (9.2)	55.5 (11.6)	**0.259**
Gender (no./no.)						
Male/female	0/58	0/81		34/13	53/9	
BMI (SD)	24.0 (3.7)	24.9 (4.0)	**0.174**	24.6 (3.5)	26.8 (21.0)	**0.489**
Comorbidities						
Diabetes	11 (19.0)	6 (7.4)*	**0.04**	9 (19.1)	16 (25.8)	**0.413**
Hypertension	8 (13.8)*	21 (25.9)	**0.083**	18 (18.3)	21 (33.9)	**0.633**
HBV	/	4 (4.9)*	**0.14**	33 (70.2)*	53 (85.5)	**0.053**
TNM						
I	/*	10 (12.3)	**0.000**	/	1 (1.6)	**0.006**
II	6 (10.3)	40 (49.4)		15 (31.9)	37 (59.7)	
III	/	12 (14.8)		4 (8.4)	6 (9.7)	
IV	52 (89.7)	19 (23.5)		28 (59.6)	18 (29.0)	
Treatment of tumor						
Un‐treatment	4 (6.9)	35 (43.2)	**0.000**	/	/	
Endocrinotherapy	7 (12.1)	3 (3.7)		/	/	
Chemotherapy	17 (29.3)	25 (30.9)		/	/	
Targeted therapy	30 (51.7)	18 (22.2)		/	/	
PD‐1 inhibitor	/	/		8 (17.0)	18 (29.0)	**0.073**
Symptomatic treatment	/	/		15 (31.9)	9 (14.5)	
Antiviral therapy	/	/		24 (51.1)	35 (56.5)	
Vaccination						
Fully	/	16 (19.8)		/	12 (19.4)	
Booster	/	65 (80.2)		/	50 (80.6)	
Severity of the COVID‐19						
Mild	49 (84.5)	77 (95.1)	**0.035**	39 (83.0)	59 (95.2)	**0.037**
Moderate	9 (15.5)	4 (4.9)		8 (17.0)	3 (4.8)	
Symptoms						
Fever	50 (86.2)	65 (80.2)	**0.359**	40 (85.1)	48 (77.4)	**0.314**
Cough	32 (55.2)	45 (55.6)	**0.964**	23 (48.9)	27 (43.5)	**0.576**
Muscle soreness	21 (36.2)*	26 (32.1)*	**0.614**	7 (14.9)	6 (9.7)	**0.405**
Sore throat	11 (19.0)	24 (29.6)	**0.153**	11 (23.4)	17 (27.4)	**0.635**
Fatigue	35 (60.3)*	39 (48.1)*	**0.155**	9 (19.1)	5 (8.1)	**0.087**
Treatments of COVID19						
Antivirals	3 (5.2)	2 (2.5)	**0.649**	/	3 (4.8)	**0.257**
Traditional Chinese medicines	30 (51.7)*	38 (46.9)*	**0.576**	7 (14.9)	13 (21.0)	**0.417**
Antibiotic	3 (5.2)	2 (2.5)	**0.649**	4 (8.5)	3 (4.8)	**0.462**
Antipyretic	31 (53.4)	52 (64.2)*	**0.203**	18 (38.3)	23 (37.1)	**0.898**
Time interval between last dose and breakthrough infection, days						
Mean (SD)	/	370.9 (109.0)		/	360.9 (149.5)	
Time interval between the breakthrough infection and blood sampling, days						
Mean (SD)	73.2 (9.7)	74.3 (13.4)	**0.988**	71.6 (18.6)	70.0 (17.1)	**0.631**

*Note*: Data are presented as No. (%) unless otherwise indicated. Chi‐Square, Fisher and *t*‐test were used. Bold values to distingush the *p* value.

* *p* < 0.05 represent the unvaccinated breast cancer versus unvaccinated liver cancer or vaccinated breast cancer versus vaccinated liver cancer.

The TNM stage of patients was considered as an important factor that leads to the vaccine hesitancy. There was no difference in the TNM staging between breast cancer and liver cancer patients who have been vaccinated; however, the number of unvaccinated patients with breast cancer at stage IV (52/58) was higher than those patients with liver cancer (28/47). In breast cancer patients, the tumor stage is closely related to the tumor‐associated therapies, while treatments do not differ in liver cancer patients.

Among the vaccinated patients, the rates of fully vaccination and the booster doses were 19.4% (12/62) and 80.6% (50/62) respectively in patients with liver cancer, and 19.8% (16/81) and 80.2% (65/81) respectively in patients with breast cancer. We also recorded symptoms after infection, medications, and the time between infection and the last vaccination. As shown that the breast cancer patients are more likely to experience muscle soreness and fatigue, and the proportions of receiving traditional Chinese medicine (Lianhua Qingwen capsules) for treatment was higher than that in liver cancer group. No patients required respiratory support or ICU admission, and the severity of COVID‐19 was significantly lower in the vaccinated patients than the vaccinated patients.

The period between the last dose and breakthrough infection in patients with liver cancer was 347, IQR (273, 414), and the period between the last dose and breakthrough in patients with cancer was 372, IQR (324, 416) days. The average time from the COVID‐19 infection to hospitalization for cancer treatment was 70.6 days for patients with liver cancer and 74.3 days for those with breast cancer (Table 1).

We conducted a further analysis of peripheral lymphocyte subsets, including CD4^+^ T, CD8^+^ T, CD19^+^ B, and NK cells, between the two groups. In patients with breast cancer, the count of CD19^+^ B cells in unvaccinated individuals was significantly higher than that in vaccinated patients, while CD4 and CD8 counts were significantly lower in unvaccinated breast cancer patients compared to vaccinated ones. For liver cancer, only the CD4 count in unvaccinated individuals was significantly lower than that in vaccinated patients. However, the CD4, CD8, and NK cell counts were even higher in breast cancer patients than in liver cancer patients in spite of vaccination status, highlighting the disparity of immune cell profiles between the two cancer groups. (Table 2).

**TABLE 2:** The distribution characteristics of lymphoid subsets in tumor populations.

	Breast cancer		*p*‐Value	Liver cancer		*p*‐Value
	Un‐vaccinated	Vaccinated		Un‐vaccinated	Vaccinated	
Lymphocyte count(/μL)	1372.1 (521.4)*	1486.8 (418.0)*	0.153	1081.3 (613.3)	1226.7 (543.2)	0.203
CD3 T cell (/μL)	900.5 (388.5)	1052.9 (330.6)*	0.014	738.4 (468.6)	858.1 (415.2)	0.171
CD3 percentage	65.1 (10.6)	72.1 (9.7)	0.000	66.3 (10.1)	69.2 (8.8)	0.125
CD4 T cell (/μL)	510.8 (247.5)	605.5 (214.2)	0.017	424.3 (272.4)	537.2 (264.9)	0.035
CD4 percentage	36.7 (9.5)	40.8 (8.9)	0.010	38.0 (9.3)	42.7 (8.7)	0.009
CD8 T cell (/μL)	348.9 (171.0)	413.1 (167.3)*	0.029	283.4 (223.0)	284.3 (181.4)	0.982
CD8 percentage	24.8 (7.3)	27.7 (8.4)*	0.039	24.9 (8.7)	22.3 (6.5)	0.084
CD4/CD8	1.6 (0.6)	1.6 (0.7)*	0.627	1.8 (1.0)	2.1 (1.0)	0.081
CD19 B cell (/μL)	199.3 (123.4)	155.6 (98.4)	0.028	189.3 (115.3)	175.7 (104.1)	0.530
CD19 percentage	14.6 (7.3)	10.2 (5.4)*	0.000	18.6 (9.9)	15.2 (7.8)	0.048
NK cell (/μL)	224.5 (138.4)*	210.2 (145.7)*	0.560	114.5 (95.3)	152.9 (118.3)	0.075
NK percentage	17.0 (9.1)*	14.1 (8.8)	0.062	11.3 (7.8)	12.9 (7.6)	0.303

*Note*: Data are presented as mean (SD) unless otherwise indicated. Fisher and *t*‐test were used.

* *p* < 0.05 represent the unvaccinated breast cancer versus unvaccinated liver cancer or vaccinated breast cancer versus vaccinated liver cancer.

### 3.2 Evaluation of anti‐spike RBD IgGs antibody level between the unvaccinated and vaccinated patients with cancer

**FIGURE 1:**
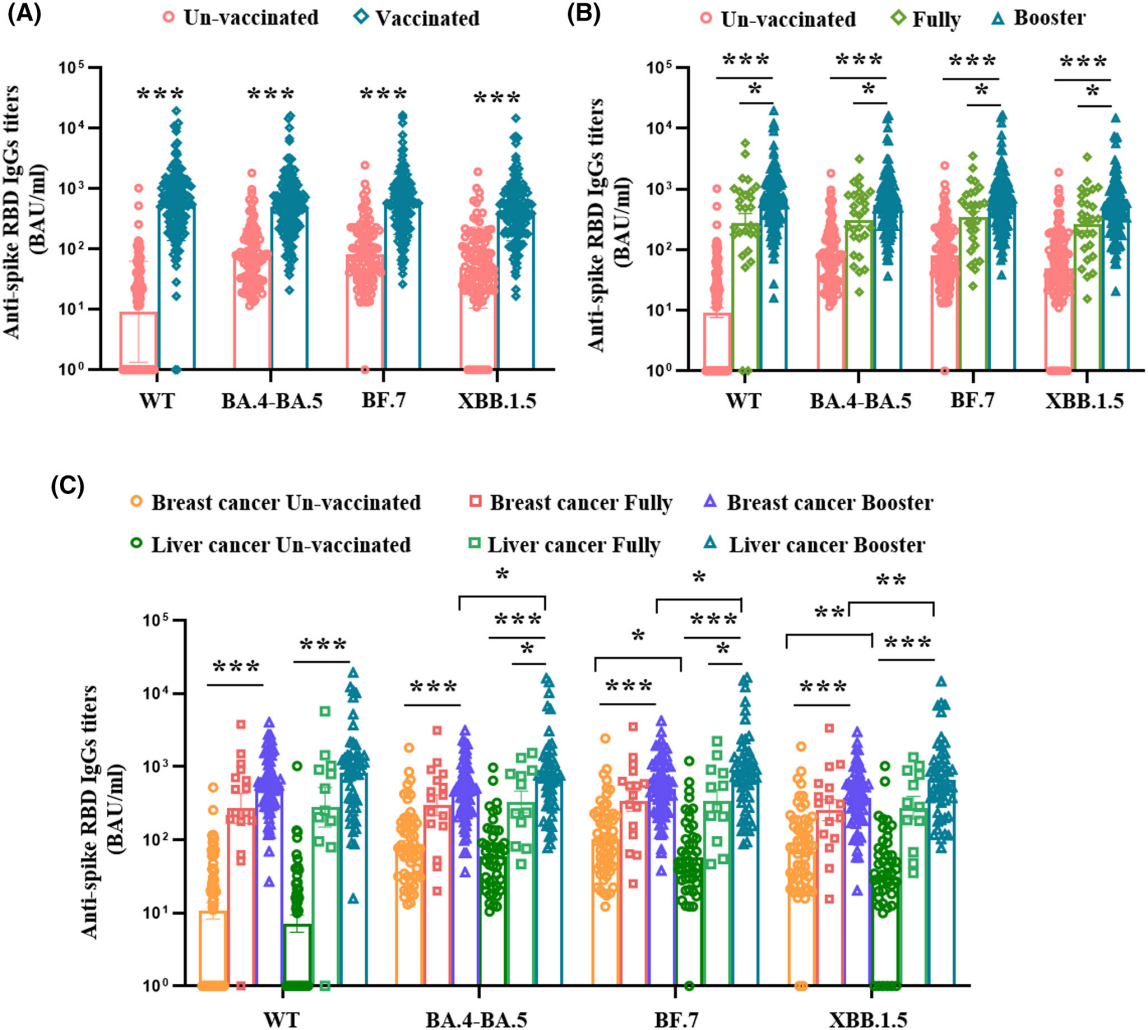
Anti‐spike receptor‐binding domain (RBD) IgGs against severe acute respiratory syndrome‐2 (SARS‐CoV‐2) variants after breakthrough infection. Vaccinated cancer patients had higher levels of anti‐spike RBD IgGs against variants than unvaccinated patients after breakthrough infection (A). The patients received fully and booster dose had significant increase of IgGs titers compared with unvaccinated patients, and booster dose further improves IgGs titers compared to fully vaccinated patients (B). Patients with liver cancer received booster dose showed higher titers against BA.4‐BA.5 and BF.7 than fully dose. For those patients received booster dose, the liver cancer group produced higher level of IgGs against BA.4‐BA.5, BF.7 and XBB.1.5 than the breast cancer group, however, the unvaccinated breast cancer group produced higher IgG of XBB.1.5 than liver cancer group (C). The *p*‐values were first analyzed using an ANOVA test. If the ANOVA test was accepted, LSD multiple comparison test was employed; otherwise, the Dunn's multiple comparison test was used. *p < 0.05, **p < 0.01, ***p < 0.001.

In this study, we compare the levels of anti‐spike RBD IgGs for the preceding (wildtype), circulating Omicron (BA.4‐BA.5, and BF.7), and new subvariants (XBB.1.5) after breakthrough infection. The vaccinated patients with cancer had higher titers of anti‐spike IgGs against WT, BA.4‐BA.5, BF.7, and XBB.1.5 than unvaccinated patients (Figure 1A), and patients who received booster doses had much higher titers than fully vaccinated patients (Figure 1B). We compared the efficacy of booster doses on the level of anti‐spike RBD IgGs in patients with different types of cancer, and found that patients with liver cancer receiving a three‐dose inactivated vaccine had the highest titers of anti‐spike RBD IgGs against WT, BA.4‐BA.5, BF.7, and XBB.1.5, within the whole population (Figure 1C). Furthermore, when patients were divided by cancer type, unvaccinated patients with breast cancer had higher levels of anti‐spike RBD IgGs against BF.7 and XBB.1.5 than patients with liver cancer after Omicron subvariant infection (*p* = 0.017, *p* = 0.005). For booster‐vaccinated patients with cancer, patients with liver cancer had significantly higher titers of anti‐spike RBD IgGs against BA.4‐BA.5, BF.7, and XBB.1.5, than patients with breast cancer after breakthrough infection (*p* = 0.013, *p* = 0.017, *p* = 0.003) (Figure 1C).

We also separately analyzed the effects of anti‐tumor treatments on the anti‐spike RBD IgGs in the two types of patients with cancer and found that vaccinated patients with breast cancer who received endocrine therapy were more prone to produce higher levels of anti‐spike IgGs against BA.4‐BA.5, BF.7, and XBB.1.5, and PD‐1 treatment had no effect on anti‐spike RBD IgG production in patients with liver cancer despite vaccination (Figure S2).

Multivariate analysis revealed that vaccine booster was the only factor associated with high titers of anti‐spike RBD IgGs against WT, BA.4, BA.5, BF.7, and XBB.1.5 in all patients with cancer (*p* < 0.05) (Table S1–S4).

### 3.3 Evaluation of neutralizing antibody titers in different patients with cancer

**FIGURE 2:**
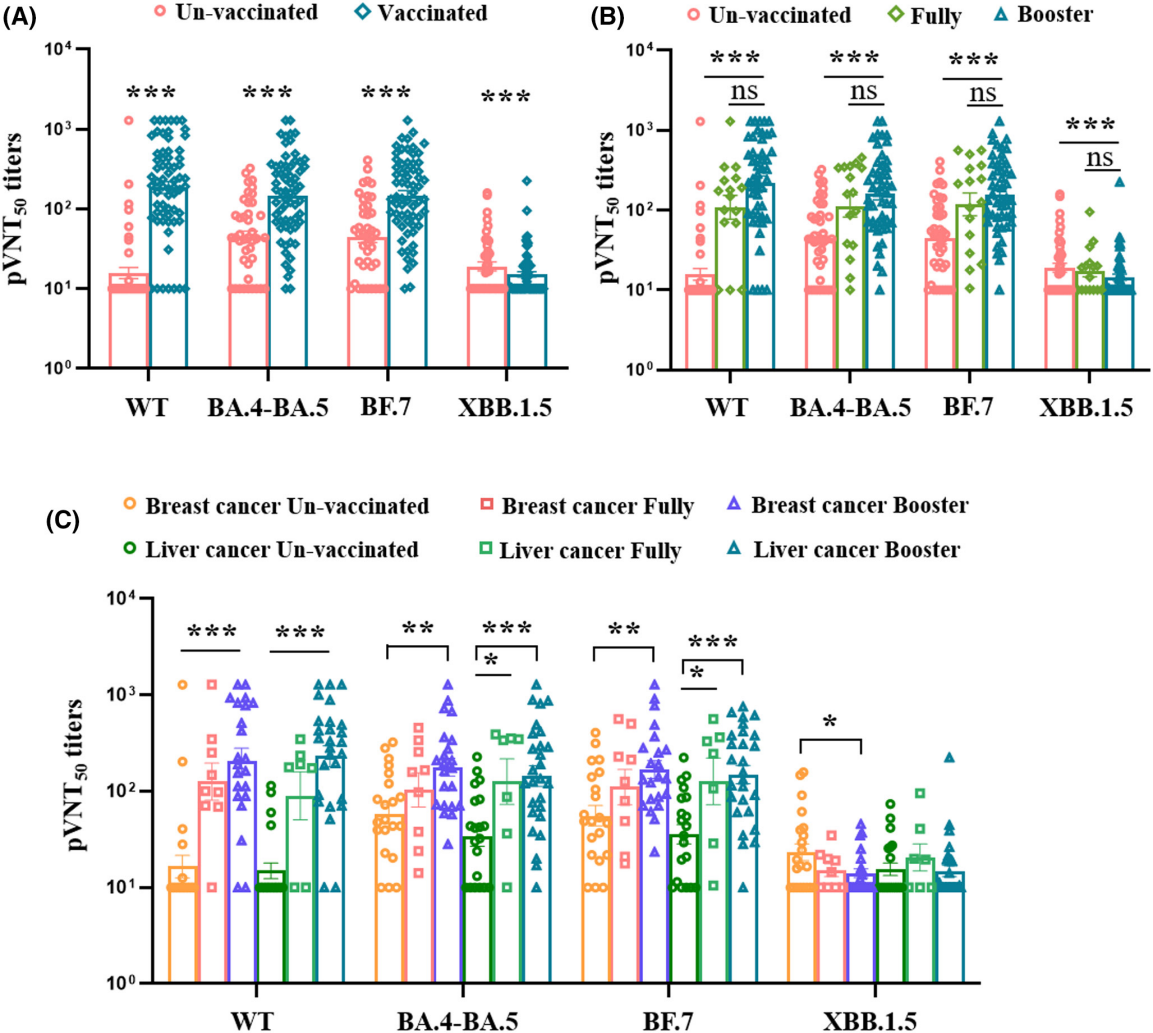
Neutralizing antibodies against SARS‐CoV‐2 variants after breakthrough infection. Vaccinated patients had a higher levels of pVNT_50_ against WT, BA.4‐BA.5, BF.7, but the unvaccinated patients had higher pVNT_50_ against XBB.1.5 than vaccinated(A). Statistical significance was observed between vaccinated and unvaccinated groups. However, receiving a booster dose did not result in a significant increase in pVNT50 levels than fully vaccination ones (B). Both cancer patients received booster vaccination produced higher levels of antibodies against WT, BA.4‐BA.5 and BF.7 than unvaccinated individuals, and only the patients with liver cancer who received a booster dose showed a significant increase in neutralizing antibodies against BA.4‐BA.5 and BF.7 compared to fully vaccinated individuals. While the unvaccinated patients with breast cancer had higher pVNT_50_ against XBB.1.5 than vaccinated breast cancer patients (C). The *p*‐values were first analyzed using an ANOVA test. If the ANOVA test was accepted, LSD multiple comparison test was employed; otherwise, the Dunn's multiple comparison test was used. * *p* < 0.05, ** *p* < 0.01, *** *p* < 0.001.

To identify the neutralizing efficacy of serum samples against SARS‐CoV‐2 variants in vaccinated patients with cancer after breakthrough infection, we first analyzed the neutralizing antibodies against wild‐type, BA.4‐BA.5, BF.7, and XBB.1.5, in unvaccinated and vaccinated patients after breakthrough infection. The results showed that vaccinated patients with cancer had higher levels of 50% pseudovirus neutralization titer (pVNT_50_) against wild‐type, BA.4‐BA.5, and BF.7 than unvaccinated patients, but the unvaccinated patients had higher titers against XBB.1.5 than vaccinated patients (Figure 2A). Second, for those patients who received a booster doses of ancestral SARSCoV‐2 produced slightly higher levels of pVNT_50_ against WT, BA.4‐BA.5, BF.7 than fully vaccined ones in spite of no significance (Figure 2B). The above results supposed that the omicron variant vaccine booster may be better than the inactivated ancestral vaccine booster for providing omicron protection.^14^ Third, we also analyzed and compared the levels of pVNT_50_ in breast cancer and liver cancer group received different dose of vaccination. In the breast cancer patients received fully vaccination did not produce higher level of pVNT_50_ against BA.4‐BA.5 and BF.7 compared to unvaccinated patients after breakthrough infection. However, pVNT_50_ levels were notably increased in patients who received a booster dose compared to unvaccinated patients (Figure 2C). Within the liver cancer group, patients who received both fully and booster doses shown a significant elevation in pVNT_50_ against BA.4‐BA.5 and BF.7 compared to unvaccinated patients (Figure 2C). Overall, all patients shown slightly higher pVNT_50_ levels after receiving a booster dose than fully vaccination. Notably, only unvaccinated breast cancer patients had a cross‐neutralization against XBB.1.5 following infection, which was not observed in patients with liver cancer (Figure 2C). These results suggested that a booster dose with the ancestral vaccine can only slightly enhance neutralizing antibody levels against the circulating Omicron variant, yet it does not confer cross‐protection against the new subvariant. (Figure 2C).

### 3.4 Association of anti‐spike RBD IgGs and neutralizing antibodies in different patients with cancer

**FIGURE 3:**
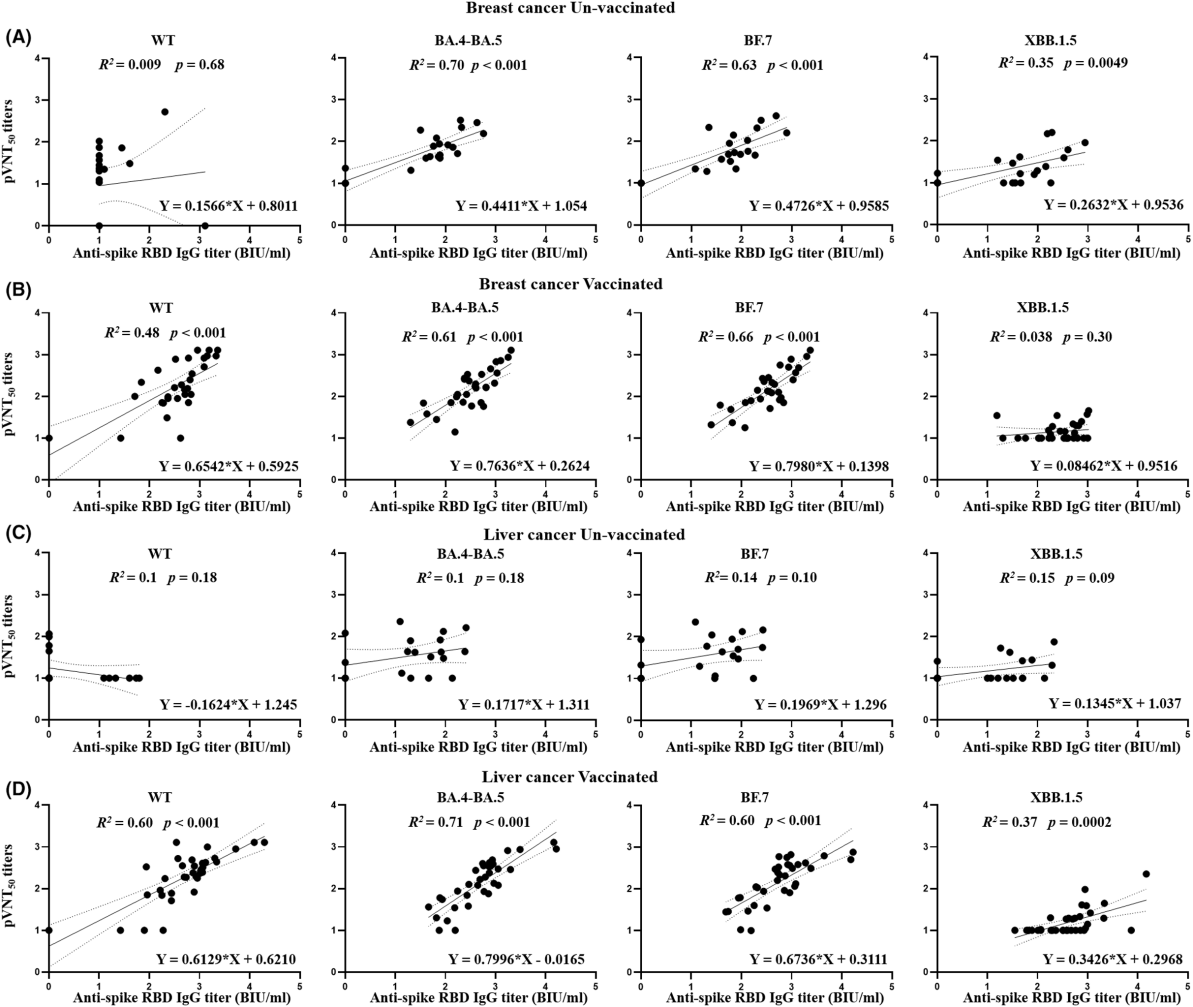
Correlations between anti‐spike RBD IgGs and 50% pseudovirus neutralization titer (pVNT_50_) among different patients with cancer. The correlations in unvaccinated patients with breast cancer (A). The correlations in vaccinated patients with breast cancer (B). The correlations in unvaccinated patients with liver cancer (C). The correlations in vaccinated patients with liver cancer (D).

The associations between the anti‐spike RBD IgGs and neutralizing antibodies (pVNT_50_) against WT, BA.4‐BA.5, BF.7, and XBB.1.5 were separately recorded among patients based on cancer type and vaccination. For unvaccinated patients, positive associations between anti‐spike RBD IgGs (BA.4‐BA.5, BF.7, and XBB.1.5) and the corresponding neutralizing antibodies were found only in unvaccinated patients with breast cancer (Figure 3A). Positive associations between anti‐spike RBD IgGs against WT, BA.4‐BA.5, and BF.7, and the corresponding neutralizing antibodies were found in all vaccinated patients after breakthrough infection (Figure 3B,D). Furthermore, a positive association between anti‐spike RBD IgG against XBB.1.5 and the corresponding neutralizing antibody was observed in vaccinated patients with liver cancer (Figure 3C). Consistent with the expected results, no association was observed between anti‐spike RBD IgG against the WT and the corresponding neutralizing antibodies in unvaccinated patients after omicron infection.

## 4 DISCUSSION

COVID‐19 inactivated vaccines, including (CoronaVac) and BBIBP‐CorV (Sinopharm), are designed to act against ancestral SARS‐CoV‐2 and are widely used in China.^9^, ^30^ During the surge of omicron variant infections in the winter of December 2022 in Beijing, a vaccine booster was identified to alleviate disease severity, shorten the time of nucleic acid conversion, and help reduce mortality in high‐risk populations in our previous study.^31^, ^32^ While the safety of COVID‐19 vaccines in various cancer types has been confirmed, the differences in antibody characteristics after infection among different types of cancer are not yet fully understood.^18^ In addition, the effectiveness of neutralizing antibodies against SARS‐CoV‐2 variants has not been identified in non‐vaccinated or vaccinated patients with cancer after omicron variant infection.

During the omicron variant breakthrough infection, booster doses elicited higher levels of anti‐spike RBD IgGs against BA.4‐BA.5, and BF.7 in patients with liver cancer than in fully vaccinated patients with breast cancer. The results indicated that patients with liver cancer are more likely to be activated by booster doses, and sex differences between patients may lead to the disparity.^18^, ^33^ In addition the higher percentage of CD19^+^ B in patients with liver cancer than in patients with breast cancer may account for the better response against circulating omicron (Figure S3).^6^, ^10^

For those liver cancer patients received an ancestral inactivated vaccine as a booster, increased anti‐spike RBD IgGs against wild‐type, BA.4‐BA.5, and BF.7, and cross‐reactivity to XBB.1.5 did not allow omicron neutralization in the pVNT_50_ analysis, indicating that the anti‐spike RBD IgGs produced in patients with liver cancer may be dysfunctional. Several factors may have accounted for this discrepancy. First, most patients with liver cancer have hepatitis B virus infection, and patients with chronic liver disease have a lower immunologic response to SARS‐CoV‐2 vaccines.^34^ Specifically, the lower number of CD3^+^T cells and a higher ratio of CD4/CD8 in vaccinated patients with liver cancer suggest that the un‐coordinated immune response may impair the adaptive immune response to omicron.^35^ Second, neutralizing antibodies against viruses may be influenced by tumor‐associated treatments and tumor disease severity. Third, the tumor‐associated microenvironment, including inflammatory cytokines and chemokines, may play a role in antibody production or maturation; therefore, it is unreasonable to use anti‐spike RBD IgGs to evaluate the efficacy of vaccines, and the potential threshold for protection may differ according to the type of cancer. Considering the demographic characteristics and underlying diseases, the interval between vaccine regimens should be optimized for patients with cancer, especially during emerging epidemic outbreaks.

Most trials of COVID‐19 vaccines excluded patients with active malignancies; however, during the surge in omicron, patients with cancer who refused vaccination were inevitably infected. Neutralizing antibodies are known to be higher in vaccinated patients and can reduce disease severity. Notably, in unvaccinated patients with cancer, we found that patients with breast cancer have higher levels of anti‐spike RBD IgGs against BF.7 and XBB.1.5 than patients with liver cancer after omicron infection. The higher number of NK cells in unvaccinated patients with breast cancer may contribute to coping with the primary infection, highlighting the importance of immunological profiles between the two types of patients with cancer after omicron infections.^36^

Additionally, unvaccinated patients with breast cancer produce higher levels of neutralizing antibodies against XBB.1.5 than patients vaccinated against ancestral SARS‐CoV‐2, indicating that the vaccine against ancestral SARS‐CoV‐2 may interfere with the cross‐reactivity caused by the circulating omicron.^37^ Therefore, omicron infection may preferentially trigger ancestral vaccine‐related cross‐reactions in vaccinated patients, whereas omicron‐related cross‐reactions may be triggered in unvaccinated patients. Therefore, we recommend evaluating immunological profiles before vaccinating against the new viral variants, and boosters containing new SARS‐CoV‐2 immunogens should be considered for vaccinated patients.^12^, ^14^

This study has some limitations. The sampling limitation might be a confounding factor influencing the results. Furthermore, most enrolled patients had liver and breast cancer, and the neutralization profiles of serum samples from patients with other types of cancer were not investigated. Furthermore, this is only a sectional study, and another follow‐up study is needed to investigate the dynamics of neutralizing antibodies in different patients with cancer. We cannot ignore the emergence of new viral variants; therefore, the protection of COVID‐19 vaccines needs to be cautioned, and more efforts are needed to promote the development of efficient vaccines.

Our study demonstrated that vaccinated patients with cancer accepted booster doses of ancestral SARS CoV‐2 before circulating omicron breakthrough infection produced higher titers of anti‐spike IgGs against WT, BA.4‐BA.5, BF.7 and XBB.1.5 than unvaccinated patients with cancer. These results suggest that the vaccine was effective. The booster dose in patients with liver cancer did not cause higher omicron neutralization than in patients with breast cancer despite higher IgG production, which may be because of un‐coordinated natural and adaptive immune system in patients with liver cancer. Therefore, different vaccine strategies should be optimized considering the immunologic profiles of different patients with cancer. Furthermore, breakthrough infection in vaccinated patients with cancer has no benefit in inducing a substantial boost in neutralizing activity against new subvariants compared to that in unvaccinated patients, indicating that the ancestral SARS‐CoV‐2 vaccine may interfere with the immunological response against new subvariants during the surge of omicron. Therefore, vaccine components against new subvariants are recommended for all patients, particularly vaccinated patients.

## AUTHOR CONTRIBUTIONS

**Shaohua Zhang:** Data curation (equal); formal analysis (equal); investigation (equal). **Lili Tang:** Formal analysis (equal); software (equal); visualization (equal). **Chunmei Bao:** Data curation (equal); formal analysis (equal); investigation (equal); methodology (equal). **Siyu Wang:** Data curation (equal); investigation (equal). **Bo Li:** Investigation (equal). **Lei Huang:** Investigation (equal). **Hua Song:** Investigation (equal). **Junliang Fu:** Investigation (equal). **Zhe Xu:** Investigation (equal). **Fanping Meng:** Investigation (equal). **Lin Cao:** Investigation (equal). **Yingying Gao:** Investigation (equal). **Yue Yuan:** Investigation (equal). **Yangliu Chen:** Investigation (equal). **Jinhong Yuan:** Investigation (equal). **Chunbao Zhou:** Investigation (equal). **Fan Li:** Investigation (equal). **Lili Qin:** Investigation (equal). **Yingfei Guo:** Investigation (equal). **Chao Zhang:** Writing – review and editing (equal). **Jinwen Song:** Writing – review and editing (equal). **Xing Fan:** Writing – review and editing (equal). **Zefei Jiang:** Writing – review and editing (equal). **Fu‐Sheng Wang:** Funding acquisition (equal); methodology (equal); project administration (equal); writing – review and editing (equal). **Ruonan Xu:** Conceptualization (equal); funding acquisition (equal); project administration (equal); writing – original draft (equal).

## FUNDING INFORMATION

The study was jointly supported by the Emergency Key Program of Guangzhou Laboratory (No. EKPG21‐30‐4), the National Key Research and Development Plan (No. 2022YFC2304404, No. 2022YFC2304803), and the Military Emergency Research Project on COVID‐19 (No. BWS20J006).

## CONFLICT OF INTEREST STATEMENT

The manuscript has been read and approved by all the authors, no conflicts of interest.

## Supporting information


Figure S1.

Figure S2.

Figure S3.

Table S1.

## Data Availability

The data that support the findings of this study are available from the corresponding author upon reasonable request.
