# Supplementary figures and images for: Omicron neutralization character in patients with breast cancer and liver cancer after the nationwide omicron outbreak
Source: Cancer Med. 2024 Jun 3;13(11):e7304. doi: 10.1002/cam4.7304 (PMC11144947; doi:10.1002/cam4.7304)

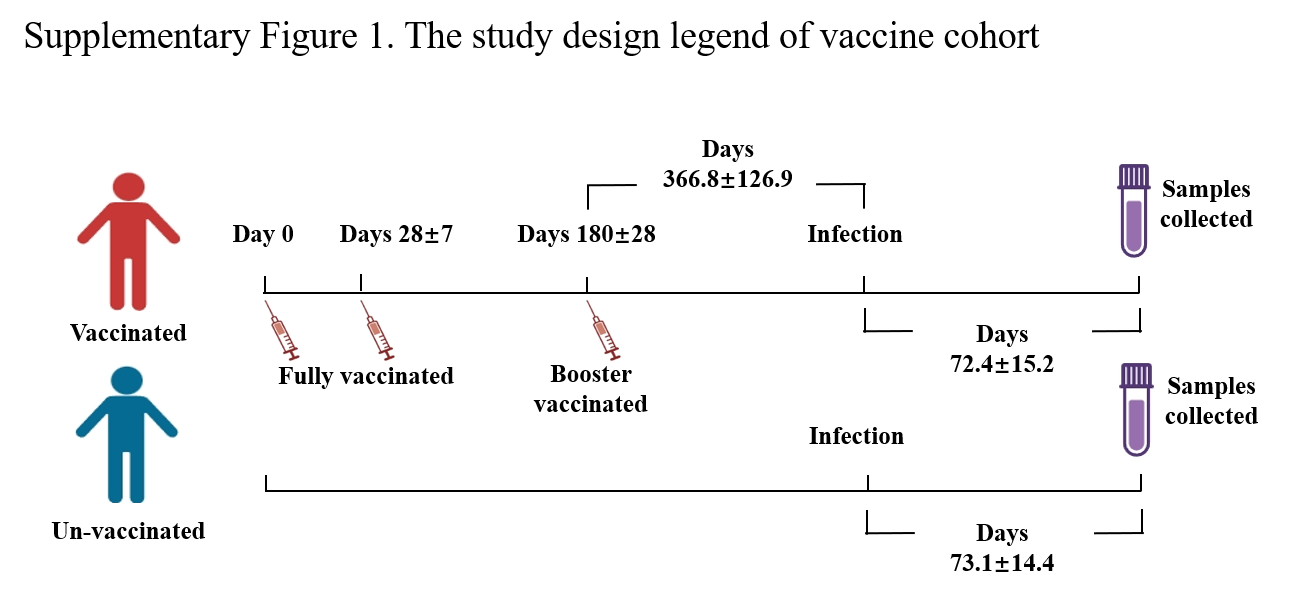

Supplement: Supplementary file 1 — Figure S1. [file CAM4-13-e7304-s002.tif]

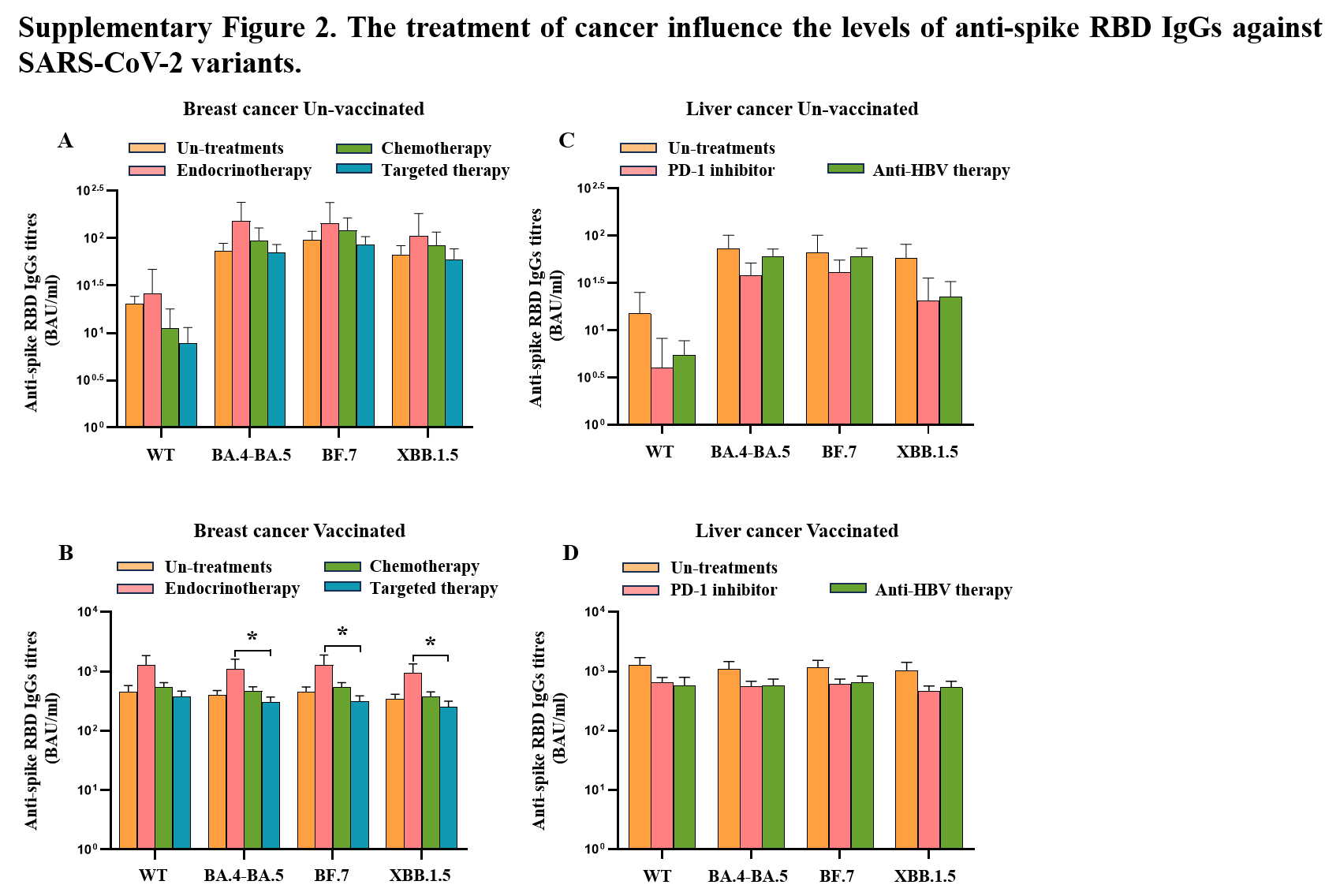

Supplement: Supplementary file 2 — Figure S2. [file CAM4-13-e7304-s001.tif]

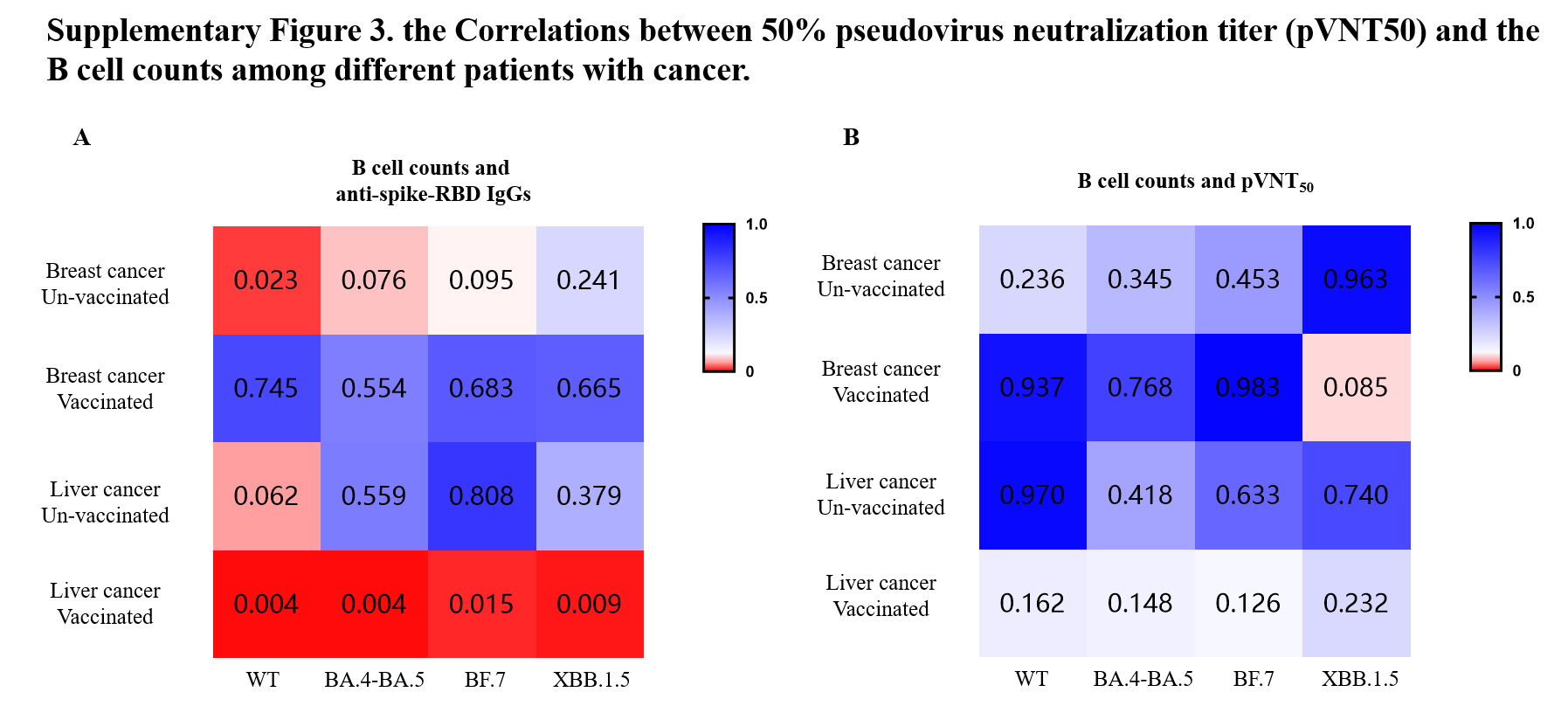

Supplement: Supplementary file 3 — Figure S3. [file CAM4-13-e7304-s004.tif]
