# Supplementary material for: Omicron neutralization character in patients with breast cancer and liver cancer after the nationwide omicron outbreak
Source: Cancer Med. 2024 Jun 3;13(11):e7304. doi: 10.1002/cam4.7304 (PMC11144947; doi:10.1002/cam4.7304)
Supplement: Supplementary file 4 — Table S1. [file CAM4-13-e7304-s003.docx]

**Supplementary table 1. Multivariable liner regression of Anti-ancestral spike-RBD-IgGs in different cancer patients**

| **Cancer types** | **Factor** | **B** | **β** | ***p*** | **F** | ***R^2^*** |
| --- | --- | --- | --- | --- | --- | --- |
| **Breast cancer** | constant | 1.059 |  | 0.000 | 202.951*** | 0.594 |
|  | dose | 0.573 | 0.773 | 0.000 |  |  |
| **Liver cancer** | constant | 0.884 |  | 0.000 | 188.398*** | 0.634 |
|  | dose | 0.687 | 0.799 | 0.000 |  |  |
| ****p*<0.001 |  |  |  |  |  |  |

The following variables were included in the multivariable liner regression model, using upward selection: age, BMI, comorbidities, treatments of tumor, does, tumor node metastasis (TNM) stage.

**Supplementary table 2. Multivariable liner regression of Anti-BA.4&BA.5 spike-RBD-IgGs in different cancer patients**

| **Cancer types** | **Factor** | **B** | **β** | ***p*** | **F** | ***R^2^*** |
| --- | --- | --- | --- | --- | --- | --- |
| **Breast cancer** | constant | 1.382 |  | 0.000 | 45.073*** | 0.390 |
|  | dose | 0.242 | 0.610 | 0.000 |  |  |
|  | age | 0.011 | 0.197 | 0.004 |  |  |
| **Liver cancer** | constant | 1.783 |  | 0.000 | 117.094*** | 0.518 |
|  | dose | 0.361 | 0.723 | 0.000 |  |  |
| ****p*<0.001 |  |  |  |  |  |  |

The following variables were included in the multivariable liner regression model, using upward selection: age, BMI, comorbidities, treatments of tumor, does, tumor node metastasis (TNM) stage.

**Supplementary table 3. Multivariable liner regression of Anti-BF.7 spike-RBD-IgGs in different cancer patients**

| **Cancer types** | **Factor** | **B** | **β** | ***p*** | **F** | ***R2*** |
| --- | --- | --- | --- | --- | --- | --- |
| **Breast cancer** | constant | 1.486 |  | 0.000 | 40.539*** | 0.364 |
|  | dose | 0.233 | 0.591 | 0.000 |  |  |
|  | age | 0.010 | 0.188 | 0.006 |  |  |
| **Liver cancer** | constant | 1.890 |  | 0.000 | 59.032*** | 0.518 |
|  | dose | 0.378 | 0.709 | 0.000 |  |  |
|  | complications | -0.204 | -0.134 | 0.048 |  |  |
| ****p*<0.001 |  |  |  |  |  |  |

The following variables were included in the multivariable liner regression model, using upward selection: age, BMI, comorbidities, treatments of tumor, does, tumor node metastasis (TNM) stage.

**Supplementary table 4. Multivariable liner regression of Anti-XBB.1.5 spike-RBD-IgGs in different cancer patients**

| **Cancer types** | **Factor** | **B** | **β** | ***p*** | **F** | ***R2*** |
| --- | --- | --- | --- | --- | --- | --- |
| **Breast cancer** | constant | 1.317 |  | 0.000 | 35.41*** | 0.333 |
|  | dose | 0.243 | 0.567 | 0.000 |  |  |
|  | age | 0.010 | 0.178 | 0.012 |  |  |
| **Liver cancer** | constant | 1.495 |  | 0.000 | 118.927*** | 0.522 |
|  | dose | 0.446 | 0.726 | 0.000 |  |  |
| ****p*<0.001 |  |  |  |  |  |  |

The following variables were included in the multivariable liner regression model, using upward selection: age, BMI, comorbidities, treatments of tumor, does, tumor node metastasis (TNM) stage.
